# Risk factors and characteristics of young patients with the biliary tract carcinoma: results of a project study for biliary surgery by the Japanese Society of Hepato‐Biliary‐Pancreatic Surgery

**DOI:** 10.1002/jhbp.776

**Published:** 2020-07-30

**Authors:** Kyohei Ariake, Michiaki Unno, Hiroshi Yoshida, Shoji Kubo, Akihiko Horiguchi, Hiroki Yamaue, Masakazu Yamamoto

**Affiliations:** ^1^ Department of Surgery Tohoku University Graduate School of Medicine Sendai Japan; ^2^ Department of Surgery Iwaki City Medical Center Iwaki Japan; ^3^ Department of Hepato‐Biliary‐Pancreatic Surgery Osaka City University Graduate School of Medicine Osaka Japan; ^4^ Department of Gastroenterology School of Medicine Bantane Hospital Fujita Health University Nagoya Japan; ^5^ Second Department of Surgery Wakayama Medical University Wakayama Japan; ^6^ Department of Surgery Institute of Gastroenterology Tokyo Women's Medical University Tokyo Japan

**Keywords:** biliary tract, carcinoma, intrahepatic, cholangiocarcinoma, pancreaticobiliary maljunction

## Abstract

**Purpose:**

This study aimed to elucidate the characteristics of biliary tract carcinoma (BTC) in young patients.

**Methods:**

This is a nationwide multicenter, retrospective cohort study supervised by the Japanese Society of Hepato‐Biliary‐Pancreatic Surgery (JSHBPS). Clinicopathological data of patients aged <50 years diagnosed with BTC from January 1997 to December 2011 were collected from 211 training institutes for highly advanced surgery registered by the JHBPS.

**Results:**

Data of 774 young patients aged <50 years were obtained from 102 institutes. Pancreaticobiliary maljunction (PBM) (10.6%) was most frequently associated with young BTC. However, organic solvents caused by printing or other occupations were only 2.5%. PBM was further associated with early onset of BTC and was noted in 38.9% of patients aged <30 years. Subgroup analysis revealed that the distributions of PBM, choledochal cysts, cholelithiasis, hepatitis B virus, and past history of cancer were significantly varied depending on the site of BTC. These results suggested that each site of BTC has a different mechanism for cancer development.

**Conclusion:**

Although the most frequent factor for young BTC patients was PBM, cancer‐associated factors were dramatically different in each BTC site. These results might be useful to elucidate the etiology of young BTC patients.

## Graphical abstract

***Highlight***

Ariake and colleagues elucidated that the most frequent factor associated with biliary tract carcinoma in young patients was pancreaticobiliary maljunction. Further analysis revealed that the distribution of cancer‐associated factors differed dramatically depending on the site of biliary tract carcinoma, suggesting that each site has a different mechanism of cancer development.

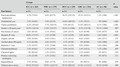

## 1 INTRODUCTION

Biliary tract carcinoma (BTC) is developed from the bile duct epithelium that arises in the liver (intrahepatic cholangiocarcinoma [ICC]), extrahepatic bile duct (perihilar cholangiocarcinoma [PHC] and distal cholangiocarcinoma [DCC]), gallbladder (gallbladder carcinoma [GBC]), and ampulla of Vater (ampullary carcinoma [AC]).^1^, ^2^ BTC is caused by the complex interaction between host‐specific genetic background and multiple environmental factors.^3^ Therefore, its incidence rates or etiology vary greatly worldwide.^3^ BTC is generally known as a disease affecting older persons (aged between 60 and 80 years), and rarely incident in younger populations.^4^, ^5^, ^6^ The Japanese cancer registry data show that from 1975 to 2013, 19 274/587 111 patients (3.3%) with gallbladder and bile duct cancer were aged <50 years.^7^

Recent reports show that exposure to organic solvents in the printing industry is strongly correlated with incidence of BTC in Japan,^8^, ^9^, ^10^ and 1.2‐dichloroethylene and dichloropropane were clarified as new carcinogenic factors.^11^ Since BTC caused by organic solvents mostly occurs in young patients,^8^, ^9^, ^10^ organic solvents are also considered to be among the major risk factors for early‐onset BTC. Previously, Kaneko et al^12^ elucidated the characteristics of occupational cholangiocarcinoma and compared them with those observed in sporadic young patients (aged <50 years). However, little is known regarding the characteristics of BTC in young patients, including its prevalence, clinicopathological features, or prognosis since it is rarely presented in this demographic.

Thus, this study aimed to investigate the characteristics of BTC in younger patients in a nationwide project study that was supervised by the Japanese Society of Hepato‐Biliary‐Pancreatic Surgery (JSHBPS).

## 2 MATERIAL AND METHODS

### 2.1 Study population

This study included patients aged <50 years (hereinafter, young patients) diagnosed with cholangiocarcinoma in training institutes with highly advanced surgery registered by the JSHBPS committee in 2014. Patients diagnosed at 50 years or above were all excluded from this study.^6^, ^12^

### 2.2 Study design

This is a nationwide, multicenter retrospective cohort study. Case report files of young BTC patients from January 1997 to December 2011 were collected after providing consent to participate in the survey.

This study evaluated three points: First, this study was conducted to elucidate the background characteristics of young BTC patients and also detect factors strongly involved in the early onset, comparing patients by age groups: 20–29, 30–39, and 40–49 years. Second, this study evaluated the distribution of cancer‐associated factors for each BTC site to understand whether the carcinogenic background was equally adapted to all sites in young BTC patients. BTC was classified according to four locations as PHC, DCC, GBC, and AC according to the Classification of biliary tract cancers established by the Japanese Society of Hepato‐Biliary‐Pancreatic Surgery: 3rd English edition.^1^ Although ICC is classified as liver cancer,^13^ we included ICC as BTC in this study because it is derived from the bile duct peripheral to the secondary branches. Therefore, BTC was subdivided into five sites, and cancer‐associated factors in each site were investigated and compared with those in other sites.

Furthermore, the biological characteristics of young BTC patients were evaluated, comparing the prognosis with the results of other nationwide study.

### 2.3 Data collection

At first, a preliminary survey was performed to investigate the total number of BTC cases and young BTC cases. Then, in the questionnaire survey, reports about young BTC cases were collected using a questionnaire made by FileMaker Pro software® Version 12 (Claris International). The survey used medical records and surgical and pathological findings. Patients or their family members were also interviewed to obtain data about past medical or occupational history.

Clinicopathological factors analyzed in this study were as follows: patient background (age, sex, date of first consultation, occupational history and duration of work, exposed substances, medical history or comorbidities, and lifestyle history), treatment methods (surgical treatments and procedure, chemotherapy, radiation therapy), pathological findings (International Union Against Cancer‐Tumor/Node/Metastasis Classification, 7th edition), and outcomes such as recurrence, date of death, or censored.

Occupations related with printing or chemical industries were as follows: printing, machinery and appliance manufacturer, machinists, chemical engineers, textile product manufacturer, synthetic resin manufacturer, medicinal chemical manufacturer, petroleum product manufacturer, and abrasive scrubber of glassware. Information about exposure to substances such as organic solvent, chemical agents, and agricultural chemicals was collected. In this study, the intensity of exposure was classified into high and low. High intensity was defined as certified occupation‐related cancer or exposure for at least 5 years, considering the result of past literature about 17 BTC patients exposed to organic solvents at least 6 years.^8^ Other patients were all defined to have low‐intensity exposure. In regard to past medical histories, this study used the already reported risk factors of BTC, with reference to past literature: hepatitis B or C infection,^14^, ^15^ liver fluke,^16^ cholecystolithiasis,^17^ choledocholithiasis,^18^ hepatolithiasis,^19^ pancreaticobiliary maljunction (PBM), ^20^ and primary sclerosing cholangitis.^21^ History of other cancer or cancer‐related disease was also surveyed.

### 2.4 Ethics

This study was approved by the Institutional Review Board Committee of the Tohoku University (Sendai, Japan) on January 28, 2013, as a supervising facility (2012‐1‐508). Approval from each institutional review board committee was also requested for each institute before participating in this study. The research was conducted in accordance with the Declaration of Helsinki.

### 2.5 Statistical analysis

All data were entered into FileMaker Pro software® (ver. 12) and exported into Microsoft Excel® (Microsoft Corp.). Statistical analyses were conducted using JMP Pro software version 14.0 (SAS Institute Inc.). Binomial variables were compared using the χ^2^ test, and continuous variables were compared using the Mann–Whitney *U* or Kruskal–Wallis test for continuous data. Overall survival (OS) rate was measured from the date of first consultation until the date of patient's death or censoring. Survival probability was calculated using the Kaplan–Meier method. *P* < .05 indicated significant difference.

## 3 RESULTS

In the preliminary survey, overall data of 36 145 BTC patients were obtained from 174 (82.5%) institutes among 211 JSHBPS training institutes. Among them, 1472 patients were identified as young patients, representing 3.8% of the entire patient population. A subsequent questionnaire survey was conducted, and 777 case reports were obtained from 102 institutes (48.3%). Among 777 case reports, three patients were 50 years old. Thus, these three patients were excluded from this study. As a result, 774 young BTC patients were identified and enrolled in this study. The median observation period from the initial consultation was 28.4 months in overall patients, 19.0 months for ICC, 30.5 months for PHC, 29.3 months for DCC, 28.9 months for GBC, and 43.3 months for AC.

### 3.1 Clinical characteristics of young BTC patients

Table 1 shows the background characteristics of the total population and each age group of BTC patients. The median age was 45 years, and the male‐female ratio was 1.40 to 1 for overall BTC patients. Among 774 patients, 162 were diagnosed with ICC, 175 with PHC, 135 with DCC, 213 with GBC, and 86 with AC. One patient was diagnosed with both ICC and GBC. This case was counted as ICC and GBC. Thus, the study analyzed 163 ICC cases and 214 GBC cases. Two patients were diagnosed with BTC based on cytological findings, but the primary site could not be detected. The BTC sites of these two patients were not included. The overall resection rate for BTC was 84.1%. With regard to the age and BTC site, AC was not found in patients in their 20s, but the rate gradually increased with age. The resection rate was not different according to the age of BTC onset among young patients.

**TABLE 1:** Characteristics of young patients with biliary tract carcinoma

	Total	Group			*P*‐value
		10–20s	30s	40s	
Number	774	18	163	594	
Age					
Median (range)	45 (15–49)	25.5 (15–29)	36 (30–39)	46 (40–49)	<.001
Sex					
Male:female	451:323	6:12	92:71	353:240	.074
Position					
ICC	21.1% (163/774)	16.7% (3/18)	27.0% (44/163)	19.6% (116/592)	.112
PHC	22.6% (175/774)	27.8% (5/18)	19.6% (32/163)	23.3% (138/592)	.527
DCC	17.4% (135/774)	38.9% (7/18)	16.6% (27/163)	17.1% (101/592)	.053
GBC	27.6% (214/774)	16.7% (3/18)	30.0% (49/163)	27.4% (162/592)	.447
AC	11.1% (86/774)	0% (0/18)	6.8% (11/163)	12.7% (75/592)	.032
Resection rate	84.1% (649/772)	83.3% (15/18)	81.0% (132/163)	84.6% (501/592)	.500

Abbreviations: AC, ampulla carcinoma; DCC, distal cholangiocarcinoma; GBC, gallbladder carcinoma; ICC, intrahepatic cholangiocarcinoma; PHC, perihilar cholangiocarcinoma.

### 3.2 Exposure factors related with each occupation

Table 2 reveals the correlation between exposure factors and occupation. High‐intensity exposure to organic solvent was found in 17 of 20 patients. Eight patients worked in the printing department, and five were machinists. Eight patients had high‐intensity exposure to chemical agents. Although two patients were chemical engineers, no particular trends were found in their occupational history. Eighteen patients were exposed to agricultural chemicals, but the work duration was not evaluated in this survey; thus, the intensity could not be defined.

**TABLE 2:** Exposure factors related to occupation

Exposure	Total	Occupation Group								
		Printing	Machinist	Machinery and appliances manufacturer	Chemical engineering	Textile product manufacturer	Abrasive scrubber of glassware	Synthetic resin manufacturer	Petroleum product manufacturer	Other
Organic solvent										
Total	20	9	5	2	1	0	0	0	0	3
High	17	8	5	2	1	0	0	0	0	1
Low	2	1	0	0	0	0	0	0	0	1
Unclear	1	0	0	0	0	0	0	0	0	1
Chemical agent										
Total	17	2	0	1	6	1	1	1	1	4
High	8	1	0	1	2	1	1	1	1	0
Low	7	1	0	0	2	0	0	0	0	4
Unclear	2	0	0	0	2	0	0	0	0	0
Agricultural chemical										
Total	18	0	0	0	1	0	0	0	0	17

### 3.3 Background characteristics of young BTC patients

Cancer‐associated factors in young BTC patients are shown in Table 3. For factors related to the exposure, only high‐intensity exposure to organic solvents and chemical agents was verified. Agricultural chemicals were not included in this analysis because of the lack of data on the exposure intensity. In this study, alcohol dose was set as 80 mg/d based on a previous review.^17^ Smoking was also analyzed at >30 pack‐years considering the results of a previous study.^22^

**TABLE 3:** Factors associated with young patients with biliary tract carcinoma

	Total	Group			*P*‐value
		10–20s	30s	40s	
Number	774	18	163	593	
Pancreaticobiliary maljunction	10.6% (82/771)	38.9% (7/18)	12.3 (20/163)	9.3% (55/591)	<.001
Choledochal cysts	4.7% (36/771)	22.2% (4/18)	6.1 (10/163)	3.7% (22/591)	<.001
Cholecystolithiasis	10.5% (81/771)	16.7% (3/18)	10.4 (17/163)	10.2% (60/591)	.671
Smoke (>30 pack–year)	10.5% (65/615)	0% (0/17)	3.0% (4/132)	13.1% (61/466)	.001
Past history of cancer	4.8% (33/686)	0% (0/17)	4.9% (7/144)	5.0% (26/524)	.643
Hepatitis B virus	4.2% (31/739)	5.6% (1/18)	2.5% (4/158)	4.6% (26/563)	.493
Organic solvent	2.5% (17/673)	0% (0/17)	4.3% (6/140)	2.1% (11/516)	.283
Alcohol abuse (>80 mg/d)	2.3% (16/689)	0% (0/18)	2.1% (3/145)	2.5% (13/526)	.771
Hepatitis C virus	2.2% (16/739)	0% (0/18)	1.3% (2/158)	2.5% (14/564)	.530
Choledocholithiasis	1.8% (14/771)	11.1% (2/18)	3.1% (5/163)	1.2% (7/591)	.003
Chemical agent	1.2% (8/673)	0% (0/17)	0.7% (1/140)	1.4% (7/516)	.742
Primary sclerosing cholangitis	1.0% (7/693)	0% (0/17)	2.0% (3/147)	0.8% (4/530)	.353
Hepatolithiasis	0.8% (6/771)	0% (0/18)	1.2% (2/163)	0.7% (4/591)	.724

Regarding factors associated with total young BTC patients, the most frequent factor was PBM (10.6%), which was subsequently found in cholecystolithiasis cases (10.5%). Exposure to organic solvents only accounted for 2.5% of all young BTC patients. Although liver fluke had been reported as one of the major risk factors for BTC in some high‐risk countries,^16^ it was not found in young Japanese patients.

In terms of the difference among each age group, the rate of smoking over 30 pack‐years was relatively low in patients in their 20s, and the rate gradually increased with age. PBM and choledocholithiasis were extracted as factors commonly found in early‐onset BTC; this finding gradually decreased with aging. Especially, PBM was dominantly correlated with BTC occurrence in patients under 30s, reaching 38.9%.

### 3.4 Patient characteristics according to the BTC site

The clinicopathological characteristics for each BTC site are shown in Table 4. The ratio of female patients was higher in GBC, at 1:1.25. The resection rate was high in AC at 97.7%; on the other hand, the resection rate in ICC was low at 72.4%. The distribution of lymph node metastasis was from 37.0% in AC to 56.8% in DCC. The rate of distant metastasis ranged from 10.7% in AC to 25.0% in ICC. Postoperative chemotherapy was administered in 47.3% of all BTC patients. It was frequently performed in ICC at 62.6%, but only 31.1% in GBC patients.

**TABLE 4:** Patient characteristics according to the area of biliary tract carcinoma

	Total	Group				
		ICC	PHC	DCC	GBC	AC
Number	774	163	175	135	214	86
Age						
Median(range)	45 (15–49)	44 (21–49)	45 (22–49)	45 (15–49)	44 (27–49)	46 (30–49)
Sex						
Male:female	451:323	97:66	123:52:00	82:53:00	95:119	55:31:00
Number of resections	84.1% (649/772)	72.4% (118/163)	80.0% (140/175)	92.4% (124/134)	85.5% (183/214)	97.7% (84/86)
TNM classification						
T						
(Tis, 1, 2, 3, 4)	38, 145, 224, 200, 129	0, 36, 59, 24, 33	0, 16, 64, 22, 59	6, 17, 20, 84, 5	30, 46, 53, 49, 30	2, 30, 28, 21, 2
N						
Positive(rate)	46.1% (333/723)	49.4% (76/154)	48.1% (75/156)	56.8% (75/132)	38.5% (77/200)	37.0% (30/81)
M						
Positive(rate)	20.6% (156/756)	25.0% (40/160)	21.8% (37/170)	13.6% (18/132)	24.3% (51/210)	10.7% (9/84)
Postoperative chemotherapy						
Yes	47.3% (301/636)	62.6% (72/115)	44.5% (61/137)	62.0% (75/121)	31.1% (56/180)	42.7% (35/82)

Abbreviations: AC, ampulla carcinoma; DCC, distal cholangiocarcinoma; GBC, gallbladder carcinoma; ICC, intrahepatic cholangiocarcinoma; PHC, perihilar cholangiocarcinoma.

### 3.5 Background characteristics according to the BTC site

Table 5 presents the distribution of cancer‐associated factors in each BTC type. The background characteristics were dramatically different at each site of BTC. The most frequent factor in ICC was hepatitis‐B virus infection (10.8%), in PHC was smoking (11.8%), in DCC was PBM (18.5%), in GBC was cholecystolithiasis (21.6%), and in AC was smoking (19.1%).

**TABLE 5:** Associated factors according to the area of biliary tract carcinoma

Past history	Groups					*P*‐value
	ICC (n = 163)	PHC (n = 175)	DCC (n = 139)	GBC (n = 214)	AC (n = 86)	
	n (%)	n (%)	n (%)	n (%)	n (%)	
Pancreaticobiliary maljunction	4.3% (7/163)	4.6% (8/175)	18.5% (25/135)	19.3% (41/213)	1.2% (1/86)	<.001
Choledochal cyst	2.5% (4/163)	2.9% (5/175)	14.8% (20/135)	3.3% (7/213)	0% (0/86)	<.001
Cholecystolithiasis	6.2% (10/162)	5.7% (10/175)	5.9% (8/135)	21.6% (46/213)	8.1% (7/86)	<.001
Smoke (>30 pack‐year)	10.5% (14/134)	11.8% (15/127)	10.4% (11/106)	6.7% (12/178)	19.1% (13/68)	.087
Past history of cancer	5.6% (8/143)	3.1% (5/161)	2.6% (3/115)	4.2% (8/190)	12.0% (9/75)	.026
Hepatitis B virus	10.8% (17/157)	1.2% (2/168)	2.3% (3/129)	3.0% (6/201)	3.6% (3/84)	<.001
Organic solvent	2.8% (4/144)	4.4% (7/161)	2.7% (3/112)	0.6% (1/183)	2.8% (2/71)	.273
Alcohol abuse (80 mg/d)	4.1% (6/148)	2.0% (3/152)	3.3% (4/120)	0% (0/188)	3.8% (3/80)	.102
Hepatitis C virus	1.3% (2/157)	4.2% (7/168)	0.8% (1/129)	2.0% (4/201)	2.4% (2/84)	.286
Choledocholithiasis	2.5% (4/162)	1.1% (2/175)	3.0% (4/135)	1.4% (3/213)	1.2% (1/86)	.695
Chemical agent	0% (0/144)	1.2% (2/161)	1.8% (2/112)	2.2% (4/183)	0% (0/71)	.344
Primary sclerosing cholangitis	1.3% (2/149)	1.2% (2/162)	2.6% (3/116)	0% (0/191)	0% (0/75)	.211
Hepatolithiasis	2.5% (4/162)	0.6% (1/175)	0.7% (1/135)	0% (0/213)	0% (0/86)	.079
Without above factor	59.5% (97/163)	70.9% (124/175)	59.3% (80/135)	51.9% (111/214)	59.3% (51/86)	.006

Abbreviations: AC, ampulla carcinoma; DCC, distal cholangiocarcinoma; GBC, gallbladder carcinoma; ICC, intrahepatic cholangiocarcinoma; PHC, perihilar cholangiocarcinoma.

The distribution of cancer‐associated factors was significantly different among five BTC sites in PBM (*P* < .001), choledochal cyst (*P* < .001), cholecystolithiasis (*P* < .001), past history of cancer (*P* = .026), and hepatitis B virus infection (*P* < .001). PBM was frequently found in DCC (n = 25; 18.5%) and GBC (n = 41; 19.3%). Choledochal cyst was specifically found in DCC (n = 20; 14.8%). Patients with AC had a history of cancer or related diseases (n = 9:12.0%); of the nine AC patients with cancer‐related medical history, three were diagnosed with familial adenomatous polyposis, one with Lynch syndrome, one with Gardner syndrome, and one with von Recklinghausen disease. Seventeen young patients had high‐intensity exposure to organic solvents, with a relatively high rate in PHC (n = 7:4.4%). However, the distribution of organic solvents could not reveal significant variance among the different BTC sites (*P* = .273).

In this study, almost 50%‐70% of patients did not have any cancer‐associated factors. On comparing the prognosis of patients with and without a cancer‐associated factor for each five BTC type, only ICC demonstrated poor prognosis in patients without cancer‐associated factor (*P* = .008) (data not shown). Further analysis demonstrated that patients without a cancer‐associated factor had a higher rate of ≥ T3 (72.6% vs 55.1%: *P* = .029), N1 (57.8% vs 36.5%: *P* = .010), and M1 (31.6% vs 15.6%: *P* = .023) in ICC. These data revealed that achieving early diagnosis in ICC patients without cancer‐associated factor was difficult.

### 3.6 Prognosis of young BTC patients

**FIGURE 1:**
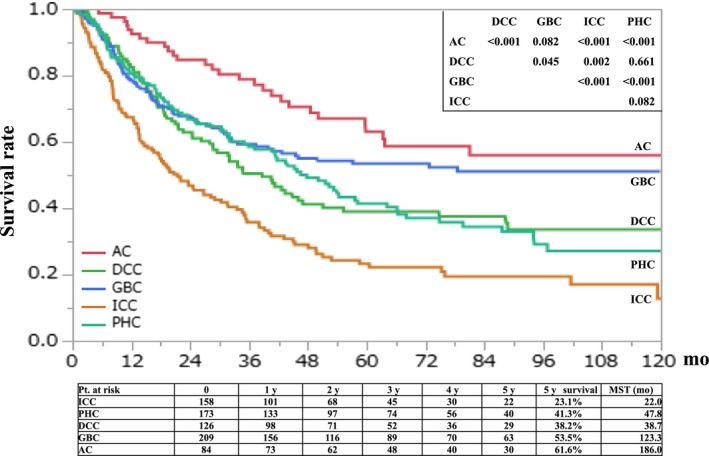
Overall survival of young BTC patients. AC, ampulla carcinoma; DCC, distal cholangiocarcinoma; GBC, gallbladder carcinoma; ICC, intrahepatic cholangiocarcinoma; PHC, perihilar cholangiocarcinoma

In this study, 5‐year OS rates were evaluated from the initial consultation (Figure 1). As a result, 5‐year OS rates and median survival time were 23.1% and 22.0 months in ICC, 41.3% and 47.8 months in PHC, 38.2% and 38.7 months in DCC, 53.5% and 123.3 months in GBC, and 61.6% and 186 months in AC, respectively. The prognosis of ICC was worse compared with those in carcinoma in other sites, such as PHC (*P* < .001), DCC (*P* = .002), GBC (*P* < .001), and AC (*P* < .001). On the contrary, the prognosis of AC was better than that of PHC (*P* < .001) or DCC (*P* < .001). PHC and DCC are sometimes combined as extrahepatic cholangiocarcinoma, and the prognoses between two types were comparable in young patients (*P* = .661).

## 4 DISCUSSION

In this study, we analyzed the background characteristics of young BTC patients and found that this population was not so frequently exposed to organic solvents as we had expected. On the contrary, the most frequent factor was PBM. PBM was also deeply associated with the early onset of BTC. Further analysis on the five BTC sites revealed that cancer‐associated factors and prognosis largely varied according to each BTC site. These results suggested that each BTC was developed by site‐specific stimulant, demonstrating specific characteristics.

With respect to patient age and cancer‐associated factors, PBM is found to be the most frequent factor of early‐onset BTC. Continuous exposure of biliary tract epithelium to external stimulation is thought to be one of the major risk factors for BTC.^3^, ^17^ PBM is a congenital anatomic anomaly, and pancreatic enzymes are continuously refluxed into the bile duct.^23^ This reflux causes persistent epithelial inflammation and becomes a strong carcinogenic stimulation. This stimulation begins earlier than other environmental stimulants because PBM is a congenital anatomic anomaly. Thus, PBM is strongly involved in the early onset of BTC, and with increasing age, incidence rate with PBM patients was gradually decreased. This is presumably because the chances of exposure to other associated factors are gradually increased.

Biliary tract carcinoma is induced by several factors such as environmental stimulation, genetic background, or its combination.^3^, ^17^ This study also revealed that continuous external stimulations, such as pancreatic juice reflux, hepatitis virus‐induced infection, or biliary stone‐induced inflammation, might have an important role in the development of BTC. On the contrary, young AC patients were strongly associated with a history of another cancer or cancer‐related disease, such as adenomatous polyposis, Gardner syndrome, or Lynch syndrome. These results suggested that one of the key factors for AC is one's inherent genetic background. Concerning the onset of AC, AC could not be found in patients in their 20s but was gradually appearing in their 30s. This result suggested that young AC patients showed relatively slow development, with relatively weak stimulation promoting cancer development. Taken together, AC is influenced by genetic predisposition and develops over a certain period of time. These characteristics were different from those of other BTC sites. Considering these results, carcinoma‐stimulated factor or development mechanisms were different and dependent on the site of BTC in young patients.

A recent study revealed that 1.2‐dichloropropane and dichloromethane, which are organic solvents used in the printing industry, induced BTC especially in young patients.^9^, ^10^ The results of that study suggested that exposure to organic solvents or a printing‐related occupation may be among the most important carcinogenetic factors in the development of BTC in young patients. This nationwide study was designed to test this hypothesis. Our results showed that most young BTC patients were working in the printing industry (Table S1), and the exposure factor was organic solvent as the previous studies demonstrated.^9^, ^10^ However, the results of this study demonstrated that organic solvents only accounted for 2.5% of the total number of young BTC patients. This is because the number of workers who had been exposed to a high concentration of 1,2‐dichloropropane and/or dichloromethane for a long time was limited. Furthermore, the proportion of exposure to organic solvents might be gradually decreased in the near future because the correlation between the printing industry and BTC incidence rates is well known, and preventive activity has already been undertaken. ^24^ Taken together, organic solvents would not remain as a major contributor to the early onset of BTC.

Past studies also suggested that detailed analysis of occupational histories might help detect unknown factors related with BTC. Based on these findings, this nationwide study also surveyed patients' occupational history and exposed factors. Regarding new potential risk factors, this study focused on eight patients with high‐intensity exposure to chemical agents and 18 patients with exposure to agricultural chemicals. The exposure intensity of agricultural chemicals could not be assessed in this study. Thus, this study could not describe the possibility of agricultural chemicals as new potential risk factors for BTC. Although 6 of 18 patients had other BTC‐associated factors, 12 patients were diagnosed with BTC without any other remarkable background characteristics, except for their exposure to agricultural chemicals. Taken together, other unknown carcinogenic factors might have existed in agricultural or chemical agents. Further studies are required to identify new carcinogenic factors by thoroughly investigating these occupations or exposure factors.

The prognosis of young BTC patients was compared with that reported in other nationwide studies conducted in Japan to evaluate the prognostic characteristics of young BTC patients (Table 6).^4^, ^5^ PHC and GBC revealed better prognosis in young BTC patients. For GBC, this study included many patients with early‐stage cancer such as carcinoma in situ. This population had improved total resection rate of GBC and prolonged survival without adjuvant chemotherapy. Thus, this study demonstrated favorable prognosis of GBC in young patients compared to another nationwide study.^4^

**TABLE 6:** Prognosis and progression of biliary tract carcinoma in young patients in comparison with other nationwide study results

	Author	Publication year	Study period	Number	Resection rate	≥T3 (%)	N1 (%)	M1(%)	5‐y OS
ICC	Kudo et al	2019	1998–2009	4436	54.4%	n.d	n.d	n.d	28.9%
	Young Case		1997–2011	163	72.4%	37.5%	49.4%	25.0%	23.1%
PHC	Ishihara et al	2016	2008–2013	2406	87.0%	35.1%	22.7%	12.1%	24.2%
	Young case		1997–2011	175	80.0%	50.3%	48.1%	21.8%	41.3%
DCC	Ishihara et al	2016	2008–2013	4091	92.9%	60.2%	28.1%	6.3%	39.1%
	Young case		1997–2011	135	92.4%	67.4%	56.8%	13.6%	38.2%
GBC	Ishihara et al	2016	2008–2013	4534	72.9%	34.1%	18.7%	18.6%	39.8%
	Young case		1997–2011	214	85.5%	38.0%	38.5%	24.3%	53.5%
AC	Ishihara et al	2016	2008–2013	2161	95.0%	30.0%	23.6%	6.9%	61.3%
	Young case		1997–2011	86	97.7%	27.7%	37.0%	10.7%	61.6%

Abbreviations: AC, ampulla carcinoma; DCC, distal cholangiocarcinoma; GBC, gallbladder carcinoma; ICC, intrahepatic cholangiocarcinoma; PHC, perihilar cholangiocarcinoma.

Although the resection rate was relatively poor and the clinical status at diagnosis was far advanced, PHC demonstrated better prognosis. The subgroup analysis for PHC patients revealed that the 5‐year survival rate of N1 or M1 cases was similar to that of past literature (Table S2). In contrast, the survival rate of N0 patients was dramatically prolonged in young patients compared with that of past literature (66.5% vs 33.9%). Although only 51.9% of N0 cases occurred in all the young patients, this improvement in prognosis was considered to contribute to their prolonged survival rate. There might be several reasons for the better prognosis in young N0 patients; however, the only method to achieve long‐term prognosis in PHC is surgical resection.^25^ The standard surgical procedure for PHC is major hepatectomy, which is sometimes associated with a high mortality rate of approximately 5% in Japan^26^, ^27^, ^28^ to 15% in other countries.^28^ Compared with older patients, young patients might be able to survive its severe postoperative period. Thus, aggressive surgery to achieve curative resection might be well performed in young patients. However, this study could not collect data in patients aged >50 years; thus, we could not draw a robust conclusion. Further analysis is needed to understand the mechanisms of better prognoses in young PHC patients.

Considering the timing of diagnosis, young BTC patients showed relatively advanced stage, including frequent lymph‐node metastasis or distant metastasis. In particular, achieving early diagnosis in young ICC patients is difficult if they did not have any cancer‐associated factor. However, the prognosis of patients with ICC, DCC, or AC demonstrated comparable or slightly worse prognosis than reported in other nationwide studies. This discrepancy could not explain the effect of surgical resection or related outcome. Further studies are needed for better prognosis in young patients even if they have far advanced staging.

This study has several limitations. First, this study collected data from half of the JSHBPS training institutes. Thus, data could not necessarily represent the entire country. This limitation leads to institutional bias. Second, this is a retrospective study using questionnaire survey for data collection. Therefore, there are some missing data for the analysis. Finally, this study did not collect data of BTC patients aged >50 years, since the analysis was performed only among younger patients. Thus, the data on the prognosis and cancer‐associated factors could not be confirmed as specific characteristics for young patients.

To the best of our knowledge, this is the largest study about young BTC patients aged <50 years. This study elucidated that each site of BTC has different background characteristics, which might be useful to elucidate the cancer development mechanism of BTC. Moreover, these results suggested that if further studies concerning the etiology or epidemiology for young BTC are planned, they should be performed for each BTC type. Especially, PHC should be differentiated from DCC to distinctly identify their incidence in young patients, though they are sometimes combined as extrahepatic BTC.^29^

In conclusion, this study elucidated that organic solvents were relatively minor carcinogenic factors regarding the early onset of BTC in young patients and PBM was the most frequent cause. Background characteristic or prognosis varied depending on site of BTC in young patients, indicating that each BTC site has different mechanisms of cancer development. Thus, further studies investigating the etiology of at each BTC site in young patients should be performed.

## CONFLICT OF INTEREST

The authors declare that they have no conflicts of interest to disclose.

## Supporting information

Table S1‐S3
