# Supplementary material for: Risk factors and characteristics of young patients with the biliary tract carcinoma: results of a project study for biliary surgery by the Japanese Society of Hepato‐Biliary‐Pancreatic Surgery
Source: J Hepatobiliary Pancreat Sci. 2020 Jul 30;27(9):571–80. doi: 10.1002/jhbp.776 (PMC7540267; doi:10.1002/jhbp.776)
Supplement: Supplementary file 1 — Table S1‐S3 [file JHBP-27-571-s001.docx]

**Supplemental Table 1: Occupational history of young biliary tract carcinoma patients**

| Occupational history |  | Group | | | | |
| --- | --- | --- | --- | --- | --- | --- |
|  | Total | ICC | PHC | DCC | GBC | AC |
| Printing | 15 | 3 | 9 | 3 | 0 | 0 |
| Machinery and appliances manufacturer | 14 | 5 | 4 | 0 | 3 | 2 |
| Machinist | 9 | 2 | 2 | 1 | 2 | 2 |
| Chemical engineering | 7 | 1 | 1 | 1 | 4 | 0 |
| Textile product manufacturer | 4 | 1 | 2 | 1 | 0 | 0 |
| Synthetic resin manufacturer | 4 | 1 | 0 | 1 | 1 | 1 |
| Medicinal chemicals manufacturer | 1 | 1 | 0 | 0 | 0 | 0 |
| Petroleum products manufacturer | 1 | 0 | 0 | 0 | 1 | 0 |
| Abrasive scrubber of glassware | 1 | 0 | 1 | 0 | 0 | 0 |

AC, ampulla carcinoma; DCC, distal cholangiocarcinoma; GBC, gallbladder carcinoma; ICC, intrahepatic cholangiocarcinoma; PHC, perihilar cholangiocarcinoma

**Supplemental Table 2: Prognosis of N0, N1, and M1 in young perihilar cholangiocarcinoma patients in comparison with other nationwide study results**

| Authors | Publication year |  | N0 | N1 | M0 |
| --- | --- | --- | --- | --- | --- |
| Ishihara et al. | 2016 | 5-year survival  rate | 33.9%  (77.3%) | 16.7%  (22.7%) | 4.2%  (12.1%) |
| Present cohort of young patients |  | 5-year survival  rate | 66.5%  (51.9%) | 19.0%  (48.1%) | 10.7%  (21.8%) |

**Supplemental Table 3:** Board certiﬁed training institutions of Japanese Society of Hepato-Biliary-Pancreatic Surgery that registered data of young cholangiocarcinoma

| Institutions | number of patients |
| --- | --- |
| Division of Surgical oncology, Department of surgery, Nagoya university graduate school of medicine | 48 |
| Department of Hepato-Biliary-Pancreatic Surgery, Osaka City University Graduate School of Medicine | 26 |
| Department of surgery, Tohoku University Graduate School of Medicine | 26 |
| Cancer institute hospital of | 24 |
| Department of General Surgery, Chiba University | 24 |
| Hepato-Biliary-Pancreatic Surgery Division, Department of Surgery, Graduate School of Medicine, University of Tokyo | 21 |
| Department of General, Pediatric and Hepato-Biliary-Pancreatic Surgery, Kitasato University Hospital | 20 |
| Department of Surgery, Ibaraki Prefectural Central Hospital | 19 |
| Department of Surgery, Hyogo College of Medicine | 19 |
| Division of Gastroenterology, Chiba Cancer Center | 18 |
| Department of Gastroenterological Surgery, Osaka University Graduate School of Medicine | 18 |
| Department of Gastroenterological Surgery, Yokohama City University Graduate School of Medicine | 16 |
| Department of Surgery, Jichi Medical University | 15 |
| Department of Surgery, National Hospital Organization, Osaka National Hospital | 14 |
| Second Department of Surgery, Dokkyo Medical University | 13 |
| Department of Surgery, Kariya Toyota General Hospital | 12 |
| Department of Surgery, The Jikei University School of Medicine | 12 |
| Hepatobiliary-Pancreatic and Transplant Surgery, Mie University Graduate School of Medicine | 11 |
| Department of Surgery, Nagasaki University Graduate School of Biomedical Sciences | 11 |
| Division of General Surgery, Japanese Red Cross Kumamoto Hospital | 10 |
| Department of Surgery, Keio University School of Medicine | 10 |
| Division of Hepato-Biliary-Pancreatic Surgery, Department of Surgery, Kobe University Graduate School of Medicine | 10 |
| Department of Surgery, The Jikei University Kashiwa Hospital | 10 |
| Department of Surgery, Okayama Saiseikai General Hospital | 9 |
| Department of Surgery, Kansai Medical University | 9 |
| Department of Gastrointestinal Surgery, Iwate Prefectural Central Hospital | 9 |
| Department of Hepato-Biliary-Pancreatic Surgery, Tochigi Cancer Center | 9 |
| Department of surgery, Japanese Red Cross Society Himeji Hospital | 9 |
| Department of Gastroenterological and Transplant Surgery, Hiroshima University Hospital | 8 |
| Department of Surgery, Kimitsu Chuo Hospital | 8 |
| Department of Surgery, Surgical Oncology and Science, Sapporo Medical University | 8 |
| Department of surgery, Matsuyama Red Cross Hospital | 8 |
| Department of Surgery, Osaka International Cancer Institute | 8 |
| Department of Surgery, Oita Red Cross Hospital | 8 |
| Department of Surgery, Teikyo University School of Medicine | 8 |
| Department of Surgery, Kitakyushu Municipal Medical Center | 8 |
| Department of Surgery, Hokkaido P.W.F.A.C Sapporo Kosei General Hospital | 7 |
| Department of surgery, Shinshu University school of medicine | 7 |
| Division of Gastroenterological and General Surgery, St. Marianna University School of Medicine | 7 |
| Department of Surgery, Iizuka Hospital | 7 |
| Department of Gastroenterology, Fukushima Rosai Hospital | 7 |
| Division of Hepato-Biliary-Pancreatic Surgery, Department of Surgery, University of Miyazaki Faculty of Medicine | 6 |
| Department of Surgery, Japanese Red Cross Kyoto Daini Hospital | 6 |
| Department of Surgery, National Hospital Organization, Sendai Medical Center | 6 |
| Department of Surgery, Yamagata Prefectural Central Hospital | 6 |
| Department of Surgery, Saitama Medical Center, Jichi Medical University | 6 |
| Division of Digestive and General Surgery, Niigata University | 6 |
| Department of Digestive Surgery, Nihon University Itabashi Hospital | 6 |
| Division of Gastroenterological Surgery, Hyogo Cancer Center | 6 |
| Second Department of Internal Medicine, Wakayama Medical University | 6 |
| Department of Hepato-Biliary-Pancreatic Surgery, National Hospital Organization Kyushu Medical Center | 5 |
| Department of Gastroenterological, Breast and Endocrine Surgery, Yamaguchi University Graduate School of Medicine | 5 |
| Department of Gastroenterological Surgery, Akita University Graduate School of Medicine | 5 |
| Digestive Disease Center, Showa University Northern Yokohama Hospital | 5 |
| Department of Surgery, Fukaya Red Cross Hospital | 5 |
| Department of Surgery, Sagamihara Kyodo Hospital | 5 |
| Department of Surgery, Japan Community Health care Organization Osaka Hospital | 5 |
| Department of Biliary-Pancreatic Surgery, Fujita Health University | 5 |
| Department of Surgery, National Hospital Organization, Kure Medical Center and Chugoku Cancer Center | 5 |
| Department of Surgery, Nara Prefecture General Medical Center | 5 |
| Department of Gastroenterological Surgery, Iwata City Hospital | 5 |
| Department of Surgery and Science, Graduate School of Medicine and Pharmaceutical Sciences, University of Toyama | 5 |
| Department of Gastroenterological Surgery, Fukuyama City Hospital | 5 |
| Department of Gastroenterological Surgery I, Hokkaido University Graduate School of Medicine | 5 |
| Department of Gastroenterological Surgery, Kanazawa University Hospital | 4 |
| Department of Digestive Surgery, Breast and Thyroid Surgery, Kagoshima University Graduate School of Medicine and Dental Sciences | 4 |
| Department of Surgery, Tokyo Metropolitan Cancer and Infectious Diseases Center Komagome Hospital | 4 |
| Department of Surgery, Institute of Gastroenterology, Tokyo Woman’s Medical University | 4 |
| Department of Surgery, Toho University Ohashi Medical Center | 4 |
| Department of Surgery, Hachioji Digestive Disease Hospital | 4 |
| Second Department of Surgery, Hamamatsu University School of Medicine | 4 |
| Department of Surgery, Toyama Prefectural Central Hospital | 4 |
| Department of Hepato-Biliary-Pancreatic and Transplant Surgery, Fukushima Medical University | 4 |
| Department of Gastroenterological Surgery II, Hokkaido University Graduate School of Medicine | 4 |
| Department of Surgery, Anjo Kosei Hospital | 3 |
| Department of Surgical Oncology, Gifu University Hospital | 3 |
| Department of Surgery, Kumamoto Regional Medical Center | 3 |
| Department of Surgery, Japan Community Health Care Organization Ritsurin Hospital | 3 |
| Department of Digestive and General Surgery, Shimane University Faculty of Medicine | 3 |
| Department of Surgery, The Jikei University Daisan Hospital | 3 |
| Department of Surgery, Tokushima University Graduate School | 3 |
| Department of Surgery, Nara Medical University | 3 |
| Department of Clinical Oncology, Aichi Cancer Center Hospital | 2 |
| Department of Surgery, National Hospital Organization Iwakuni Clinical Center | 2 |
| Department of Surgery, National Hospital Organization Kyoto Medical Center | 2 |
| Department of General Surgical Science, Graduate School of Medicine, Gunma University | 2 |
| Department of Gastroenterological Surgery, Kagawa University School of Medicine | 2 |
| Department of Surgery, Saiseikai Suita Hospital | 2 |
| Department of Surgery, Akita City Hospital | 2 |
| Department of Surgery, Kagoshima Medical Association Hospital | 2 |
| Division of Surgery, Kasugai Municipal Hospital | 2 |
| Department of Surgery, Niigata Prefectural Central Hospital | 2 |
| Department of Surgery, Sendai Kousei Hospital | 2 |
| Department of Surgery, Funabashi Municipal Medical Center | 2 |
| Department of Surgery, National Hospital Organization Yokohama Medical Center | 2 |
| Department of Hepato-Biliary-Pancreatic Surgery, National Hospital Organization, Fukuyama Medical Center | 2 |
| Department of Gastroenterology, Musashino Red Cross Hospital | 2 |
| Department of Surgery, Ehime Prefectural Central Hospital | 1 |
| Department of Surgery, Center for Gastroenterology, Urasoe General Hospital | 1 |
| Department of Surgery, Gifu Prefectural Tajimi Hospital | 1 |
| Department of Surgery, Toyonaka Municipal Hospital | 1 |
| Department of Surgery, Chibaken Saiseikai Narashino Hospital | 1 |
